# Lack of detectable genetic isolation in the cyclic rodent *Microtus arvalis* despite large landscape fragmentation owing to transportation infrastructures

**DOI:** 10.1038/s41598-021-91824-w

**Published:** 2021-06-15

**Authors:** Julio C. Dominguez, María Calero-Riestra, Pedro P. Olea, Juan E. Malo, Christopher P. Burridge, Kirstin Proft, Sonia Illanas, Javier Viñuela, Jesús T. García

**Affiliations:** 1 IREC, Instituto de Investigación en Recursos Cinegéticos (CSIC-UCLM-JCCM), Ronda de Toledo, 12, 13071 Ciudad Real, Spain; 2 Terrestrial Ecology Group (TEG-UAM), Departamento de Ecología, Facultad de Ciencias, Universidad Autónoma de Madrid, C/Darwin 2, 28049 Madrid, Spain; 3 Centro de Investigación en Biodiversidad y Cambio Global (CIBC-UAM), Universidad Autónoma de Madrid, C. Darwin 2, 28049 Madrid, Spain; 4 Discipline of Biological Sciences, University of Tasmania, Private Bag 55, Hobart, TAS 7001 Australia

**Keywords:** Ecology, Evolution, Genetics, Molecular biology, Zoology

## Abstract

Although roads are widely seen as dispersal barriers, their genetic consequences for animals that experience large fluctuations in population density are poorly documented. We developed a spatially paired experimental design to assess the genetic impacts of roads on cyclic voles (*Microtus arvalis*) during a high-density phase in North-Western Spain. We compared genetic patterns from 15 paired plots bisected by three different barrier types, using linear mixed models and computing effect sizes to assess the importance of each type, and the influence of road features like width or the age of the infrastructure. Evidence of effects by roads on genetic diversity and differentiation were lacking. We speculate that the recurrent (each 3–5 generations) episodes of massive dispersal associated with population density peaks can homogenize populations and mitigate the possible genetic impact of landscape fragmentation by roads. This study highlights the importance of developing spatially replicated experimental designs that allow us to consider the large natural spatial variation in genetic parameters. More generally, these results contribute to our understanding of the not well explored effects of habitat fragmentation on dispersal in species showing “boom-bust” dynamics.

## Introduction

In today's globalized economy, the number of motor vehicles and the volume of transportation has reached unprecedented levels, increasing the ecological impact of the world’s human population during the last decades. Globally, roads currently cover more than a 20% of the earth’s land surface with large differences in occurrence, distribution and size of roadless areas among continents^1^. In Europe, for example, almost half of all continent is located within 1.5 km of the nearest transport infrastructure, with impacts that expand over most of the land and important reductions projected for birds and mammals^2^. Most empirical evidence suggests that this improvement of connectivity among human populations involves worsening of connectivity in wild animal populations^3–7^. In this sense, road networks can prevent animal movement, either through behavioral avoidance^8,9^ or mortality during crossing attempts^10,11^. These physical barriers may shape the dispersal behavior of species^12^, causing the isolation of populations into habitat fragments for many taxa^13–15^ with severe consequences for individuals and populations^16–18^. Research on this topic has shown that increasing habitat fragmentation and population isolation by roads can reduce locally the population size, the individual exchange among fragments and the gene flow, can have a significant impact on population genetic structure^19,20^ and often leads increased genetic drift and/or inbreeding, which can further reduce effective population sizes and genetic diversity over time. All this can have, in turn, potential implications for long-term population persistence^14,21^ due to the combined effects of inbreeding, genetic drift, and demographic and environmental stochasticity^22,23^, specially of those already small and inbred populations (but see Ref.^24^).

The barrier effect of roads varies largely with respect to road types, adjacent habitat quality, and traffic volume^25–28^. Specific road features, such as fences, median strips, drainage culverts, adjacent gravel and grassy verges, and structural passages beneath or above the paved surface influences road permeability for terrestrial species^29^. The severity of the barrier effect is often modulated by road width^27,30^ as the wider the barrier the higher the traffic intensity^13,25^, and the more reluctant or unable an individual will be to cross^31,32^. This effect is especially important in low-vagility species like small mammals or amphibians^8,33^. In addition, the time elapsed since road construction also affects the number of generations exposed to the putative barrier, influencing our ability to genetically measure the severity of the barrier effect^34–36^. However, species with high dispersal ability and abundance^34,37,38^, and large fluctuations in population size through time^39^ can mitigate the genetic effects of barriers, and our ability to detect them. Even so, studies on species that display large fluctuations in population size are particularly scarce^40,41^, limiting our understanding of the full impact of roads on animal populations.

Our ability to detect genetic effects from barriers depends also on the approach used. Studies conducted without some level of spatial replication and control groups make difficult to infer causal relationships since the results are subject to high variance caused by the interaction between species characteristics (e.g., population size, dispersal ability, habitat selection) and site-specific factors (e.g., habitat quality, road features, predation risk), which subsequently influences dispersal, relatedness and spatial genetic patterns^26,42,43^.

Here, we use the common vole *Microtus arvalis* (Pallas, 1779), a small-bodied rodent with short dispersal distances that reaches high local abundance^42,44^, as a model to test for the road effects on genetic diversity and structure in highly fragmented landscapes. The high colonization ability reported for the species in the last decades^45^ make it especially suitable to understand the dispersal mechanisms of small mammals over short time periods. Additionally, common vole populations reach high local abundances and may experience extreme fluctuations in density (1–2000 individuals per hectare)^46^ through 3–5 years population cycles with well-known phases (i.e., increase, peak, crash and low phases^47^. This cyclic variation in population size make this species particularly interesting to investigate the potential vulnerability to road barriers, compared to what has been described for other sympatric small mammals with more stable populations^8,48,49^. In a scenario of large fluctuations in population size, the time of lowest population size (low phase) should experience the highest levels of genetic drift^50,51^. As a result, demographic bottlenecks should have a strong effect on genetic variation, reducing diversity within and increasing differentiation among populations^52–54^. However, the high population size and gene flow during the peak phases could minimize the effects of drift and bottlenecks, although the system might take several generations to approach new equilibrium values^41,55,56^. In that context, investigating the effect of roads as barriers to gene flow, which can rapidly re-shuffle alleles among population demes, is particularly interesting in vole species, considering the short time lags between peak and low phases and its short generation time^57^.

In the present study, we used a spatially paired-plot design to test whether roads may influence genetic diversity and structure of populations in cyclic voles. We used study plots (2.5 × 3.0 km each) bisected by different types of paved roads (conventional 2-lane roads, 4-lane highways, or combined barriers) and spatially-matched plots bisected by an unpaved rural tracks to control for the impact of the road. We used paired t-tests, Linear Mixed-effect Models (LMMs) and estimated associated effect sizes to explore the potential barrier effect due to roads. We additionally tested if highways of different width and age (time since road construction) might influence any barrier effect. If the road is not an effective barrier, individual movements ending at the same side of the road should be equally frequent as those across the road, and therefore, genetic parameters within plots will remain unchanged. We further discuss other possible explanations to this pattern based on the characteristics of the study species, such as that the large population size reached during peak phases was marginally affected by genetic drift and there is no spatial variation in genetic structure independently of gene flow. The alternative scenario (road barrier effect) would generate—given enough generations—observable differences in the distribution of genetic variation within plots bisected by roads but not in plots without roads.

## Results

**Figure 1:**
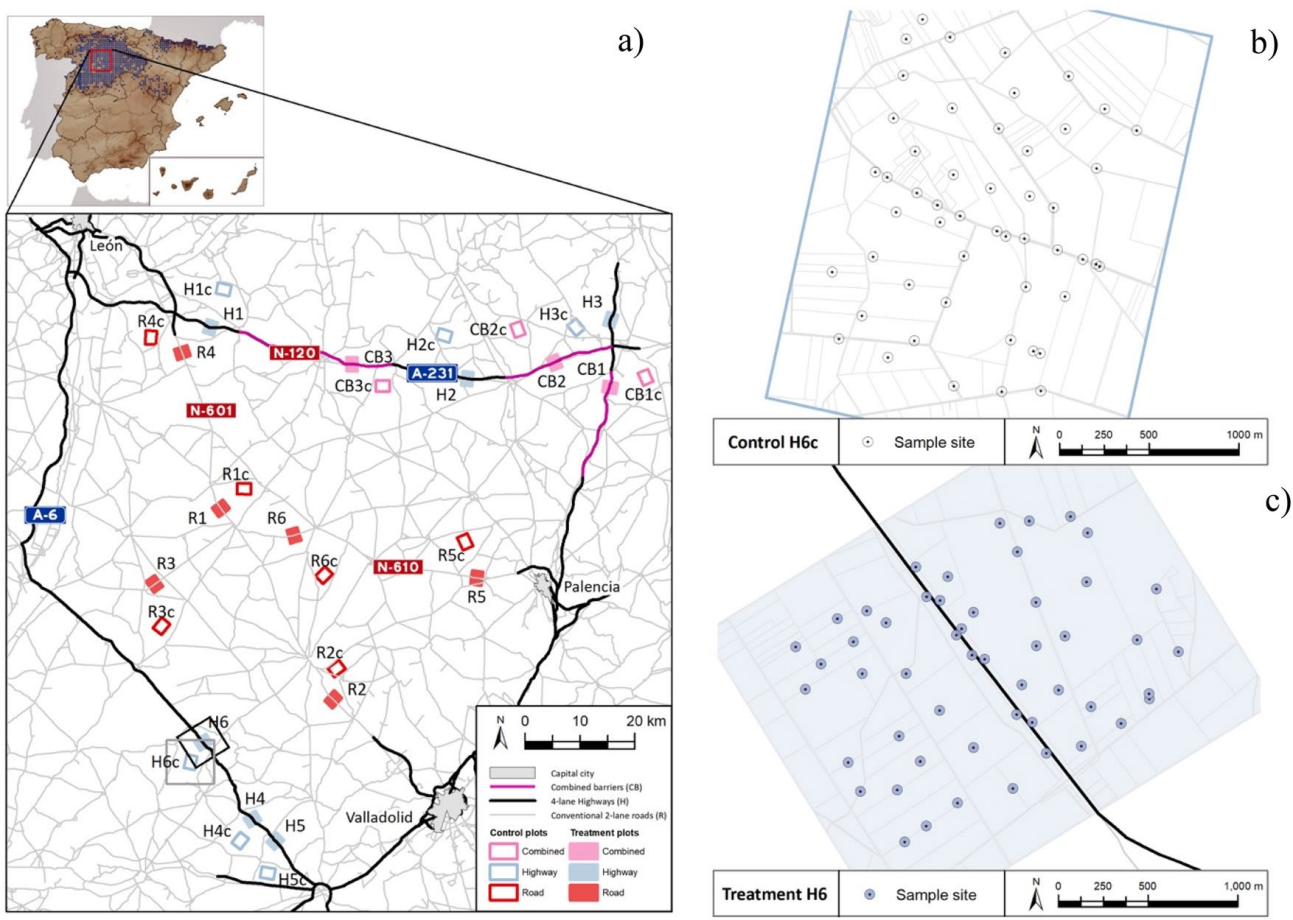
(**a**) Map of study area and sampling locations (plots). Plots are colored according to the features of the potential barrier type by which they are intersected; conventional 2-lane roads (R, Red), 4-lane highways (H, Blue), and combined barriers (CB, Purple), unpaved rural tracks (T, unfilled). Inset shows known distribution of common vole in Spain (10 × 10 km UTM-grids;^126^) where the red square delimits the study area. Plot codes correspond to Table A.1. Examples of trapping stations (10 traps per point) across control (**b**) and treatment (**c**) plots are shown for illustration purposes. Maps were generated using ESRI ArcGIS 10.3 Desktop platform (https://www.esri.com/en-us/home)^58^.

A total of 1757 common voles were sampled at 1790 different sampling points across the 30 plots (Fig. 1). Of these, 1678 specimens genotyped at 9 microsatellite loci were kept for further analyses after removing samples unsuccessfully genotyped (n = 63) or resulting in the presence of full-sibs individuals in a plot (n = 16). A summary of allelic information across all plots is shown in Table 1. Since all locus showed no departure from Hardy Weinberg equilibrium (HWE) and no evidence for null alleles across all sites (Supplementary Tables S1, S2), all of them were kept for further analysis. The variables used to describe genetic diversity, structure and differentiation of common vole are shown in Table 2.

**Table 1:** Summary of allelic information across all plots.

Locus	Allele size range (bp)	A	Ho	uHe	F_IS_	E_1_	E_2_
Ma09	72–118	24	0.864 ± 0.008	0.860 ± 0.005	− 0.016 ± 0.009	0.009	0.029
Mar102	354–422	22	0.865 ± 0.009	0.873 ± 0.004	− 0.001 ± 0.010	0.008	0.016
Mar012	77–139	28	0.851 ± 0.013	0.904 ± 0.004	0.049 ± 0.013	0.000	0.012
Mar003	142–180	20	0.884 ± 0.009	0.899 ± 0.003	0.005 ± 0.010	0.003	0.015
Mar063	270–280	7	0.345 ± 0.019	0.359 ± 0.016	0.039 ± 0.024	0.013	0.012
Mar016	160–186	14	0.799 ± 0.014	0.802 ± 0.008	− 0.007 ± 0.013	0.000	0.016
MM6	147–179	18	0.833 ± 0.011	0.852 ± 0.004	0.012 ± 0.010	0.006	0.019
Ma54	189–225	18	0.772 ± 0.014	0.814 ± 0.006	0.043 ± 0.014	0.030	0.003
Ma66	226–284	27	0.878 ± 0.010	0.891 ± 0.005	0.003 ± 0.009	0.023	0.005

Number of alleles per locus (A), observed heterozygosity (Ho), unbiased expected heterozygosity (uHe) and inbreeding coefficient (F_IS_). Allelic dropout (E_1_) and false allele rates (E_2_).

**Table 2:** Variables used in the study to describe genetic diversity, structure and differentiation of common vole.

**Genetic diversity**	
Ho	Average observed heterozygosity
uHe	Unbiased estimate of average expected heterozygosity corrected for sample size
Ar	Average allelic richness (mean number of alleles per locus adjusted for sample size)
Ho_DIF_	Ho computed as absolute differences between barrier sides
uHe_DIF_	uHe computed as absolute differences between barrier sides
Ar_DIF_	Ar computed as absolute differences between barrier sides
**Genetic structure and differentiation**	
σ_﻿bw_	σ_between_ (percentage of variance distributed between populations -individuals from opposite sides of the barrier-)/σ_within_ (percentage of variance distributed within populations,-i.e., pooled samples from same side of the barrier and considered as populations)
F_ST_	Estimator of genetic differentiation between populations (individuals from opposite sides of the barrier)
G”st	Hedrick’s standardized estimator of genetic differentiation between populations (individuals from opposite sides of the barrier)
r_Gd_	Partial Mantel text computed with codominant genotypic distance (Gd)
r_Dps_	Partial Mantel text computed with proportion of shared alleles (Dps)
r_Lrm_	Partial Mantel text computed with relatedness estimator (Lrm)

### Genetic diversity

A summary of genetic diversity parameters of study plots is presented in Table 3. Genetic diversity was high at the 9 microsatellite loci. The mean number of alleles per locus ranged from seven (locus Mar063) to 28 (locus Mar012), expected heterozygosity (uHe) from 0.36 to 0.90 (Table 1), and the average number of alleles per study plot from 9.8 (R3) to 15 (CB1; Table 3) with similar levels of heterozygosity in all study plots (uHe: 0.77–0.84).

**Table 3:** Genetic diversity estimates (mean ± se) for the studied populations (experimental and their control plots).

Plot	Na	Nb	A	Ho	He	uHe	Ar	F_IS_
CB1	47	61	15.00 ± 2.05	0.83 ± 0.04	0.83 ± 0.04	0.84 ± 0.04	11.05 ± 1.30	0.00 ± 0.01
CB1c	24	20	12.78 ± 1.87	0.82 ± 0.04	0.82 ± 0.04	0.83 ± 0.04	10.91 ± 1.40	0.00 ± 0.02
CB2	35	18	12.89 ± 1.66	0.80 ± 0.06	0.81 ± 0.06	0.82 ± 0.06	10.74 ± 1.25	0.02 ± 0.02
CB2c	20	23	12.33 ± 1.60	0.82 ± 0.05	0.82 ± 0.05	0.83 ± 0.05	10.62 ± 1.27	0.00 ± 0.02
CB3	21	18	10.89 ± 1.31	0.78 ± 0.06	0.81 ± 0.05	0.82 ± 0.05	9.87 ± 1.09	0.04 ± 0.03
CB3c	41	27	12.22 ± 1.79	0.78 ± 0.06	0.80 ± 0.06	0.81 ± 0.06	10.13 ± 1.20	0.04 ± 0.02
H1	23	20	10.67 ± 1.17	0.78 ± 0.07	0.78 ± 0.06	0.79 ± 0.07	9.44 ± 0.93	0.02 ± 0.03
H1c	27	43	11.89 ± 1.39	0.79 ± 0.08	0.79 ± 0.07	0.80 ± 0.07	9.93 ± 1.04	0.02 ± 0.03
H2	32	33	12.89 ± 1.64	0.80 ± 0.06	0.81 ± 0.06	0.82 ± 0.06	10.56 ± 1.19	0.02 ± 0.02
H2c	15	17	10.22 ± 1.13	0.81 ± 0.05	0.81 ± 0.05	0.82 ± 0.05	9.47 ± 1.00	− 0.01 ± 0.03
H3	26	27	13.67 ± 1.58	0.79 ± 0.07	0.82 ± 0.06	0.83 ± 0.06	11.47 ± 1.24	0.05 ± 0.02
H3c	33	34	12.78 ± 1.49	0.80 ± 0.05	0.82 ± 0.05	0.83 ± 0.05	10.63 ± 1.17	0.02 ± 0.02
H4	41	43	11.22 ± 1.12	0.76 ± 0.07	0.78 ± 0.07	0.79 ± 0.07	9.37 ± 0.89	0.04 ± 0.03
H4c	19	36	11.11 ± 1.22	0.76 ± 0.06	0.78 ± 0.06	0.79 ± 0.06	9.39 ± 0.93	0.04 ± 0.03
H5	46	27	11.11 ± 1.07	0.79 ± 0.05	0.80 ± 0.04	0.80 ± 0.04	9.29 ± 0.85	0.01 ± 0.03
H5c	33	22	10.56 ± 1.08	0.78 ± 0.07	0.78 ± 0.06	0.79 ± 0.06	9.26 ± 0.82	0.01 ± 0.03
H6	13	26	10.33 ± 1.13	0.78 ± 0.07	0.79 ± 0.07	0.80 ± 0.07	9.22 ± 0.94	− 0.01 ± 0.03
H6c	33	15	11.44 ± 1.20	0.76 ± 0.05	0.80 ± 0.05	0.81 ± 0.05	9.67 ± 0.90	0.06 ± 0.03
R1	23	28	11.67 ± 1.39	0.81 ± 0.05	0.81 ± 0.05	0.82 ± 0.05	10.00 ± 1.07	− 0.01 ± 0.01
R1c	33	23	11.44 ± 1.29	0.78 ± 0.07	0.78 ± 0.07	0.79 ± 0.07	9.61 ± 1.01	− 0.01 ± 0.03
R2	18	14	10.11 ± 1.21	0.77 ± 0.05	0.77 ± 0.04	0.78 ± 0.05	9.17 ± 1.04	0.00 ± 0.02
R2c	18	18	9.89 ± 0.95	0.79 ± 0.05	0.79 ± 0.06	0.80 ± 0.06	9.11 ± 0.88	− 0.02 ± 0.03
R3	19	21	9.78 ± 1.30	0.73 ± 0.08	0.76 ± 0.08	0.77 ± 0.08	8.83 ± 1.10	0.06 ± 0.03
R3c	22	29	10.67 ± 1.28	0.77 ± 0.06	0.78 ± 0.06	0.79 ± 0.06	9.41 ± 1.01	0.01 ± 0.03
R4	23	18	11.11 ± 1.03	0.80 ± 0.06	0.80 ± 0.06	0.81 ± 0.06	9.96 ± 0.93	0.01 ± 0.02
R4c	15	11	10.22 ± 1.06	0.76 ± 0.07	0.78 ± 0.06	0.80 ± 0.06	9.88 ± 1.00	0.05 ± 0.03
R5	64	30	12.11 ± 1.32	0.79 ± 0.06	0.81 ± 0.06	0.81 ± 0.06	10.07 ± 1.05	0.03 ± 0.01
R5c	72	72	13.67 ± 1.68	0.82 ± 0.06	0.82 ± 0.06	0.82 ± 0.06	10.49 ± 1.13	− 0.01 ± 0.02
R6	16	6	10.11 ± 1.18	0.83 ± 0.05	0.79 ± 0.05	0.81 ± 0.06	10.11 ± 1.18	− 0.07 ± 0.03
R6c	32	14	11.67 ± 1.31	0.78 ± 0.06	0.79 ± 0.06	0.80 ± 0.06	10.11 ± 1.09	0.02 ± 0.02

Na and Nb represent the number of samples collected at each side of the putative barrier. Average number of alleles per locus (A), observed heterozygosity (Ho), expected heterozygosity (He), unbiased expected heterozygosity (uHe), allelic richness (Ar), average inbreeding coefficient within individuals (F_IS_).

**Figure 2:**
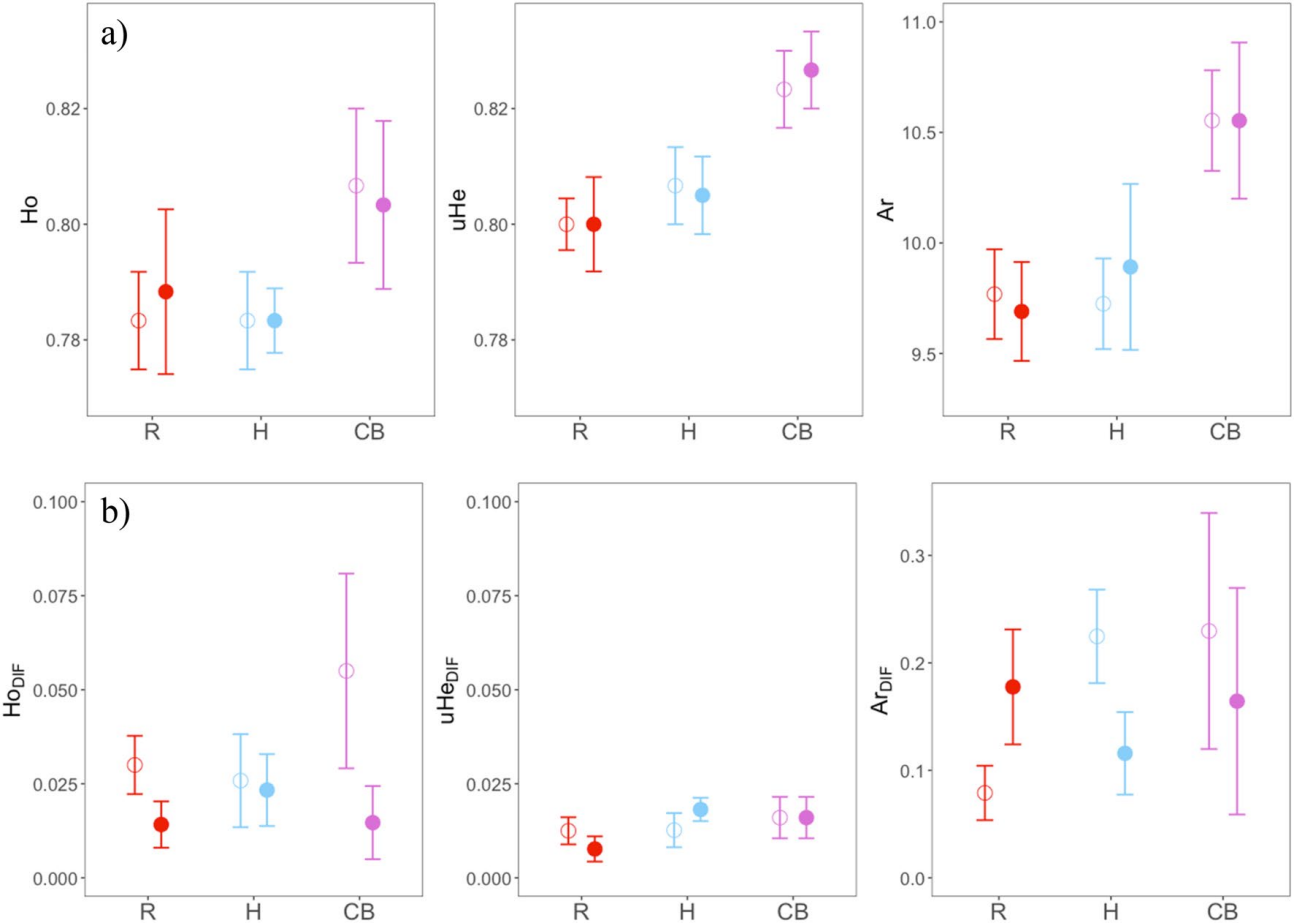
Comparison of genetic diversity estimators (mean ± s.e) between treatments (filled dots: conventional 2-lane roads R, 4-lane highways H, and combined barriers CB) and their controls (empty dots). Ho: observed heterozygosity, uHe: unbiased expected heterozygosity, A: allelic richness. Means are computed as a global mean per plot (**a**) and as an absolute difference between barrier sides (**b**).

No diversity parameters showed strong differences between experimental and control plots (paired t-test *p* value > 0.1 in all cases, with negligible to small effect sizes; Table 4). Likewise, no effect of the barrier type on genetic diversity parameters was evident from the linear mixed models (LMMs hereafter) (effect size: f^2^ ≤ 0.15; Supplementary Tables S3, S4, Fig. 2). The relationship between Ho and *Age* of the barrier was positive and the effect size was large (Cohen’s f^2^ = 1.176) while the relationship with *Ar* was negative (Cohen’s f^2^ = 1.094), indicating greater differences in observed heterozygosity and lower differences in allelic richness between plots with highways and their controls over time. However, both effect sizes were found to have 95% confidence intervals (95% CI hereafter) that includes zero, suggesting the predictors may contribute very little to the relationship. We observed no substantial effect of *Width* on these parameters (Supplementary Table S3). Roads (R) showed lower differences between barrier sides than their controls in observed heterozygosity (Ho_DIF_) while greater differences in allelic richness (Ar_DIF_). Highways (H), however, presented lower differences in allelic richness (Ar_DIF_) for experimental plots, all with large effect sizes. The strength of the relationship between *Age* and Ar_DIF_ was large and negative, and between *Width* and Ho_DIF_ was large and positive (Supplementary Table S4), indicating that differences in allelic richness between barrier sides among experimental and control plots diminished over time whereas differences in observed heterozygosity increased with increasing width, but as in the previous cases 95% CI included zero and was too wide to represent an acceptable level of uncertainty. We also discarded the existence of correlation between these differential estimators and sample size per plot (Ho_DIF_; r = 0.202, uHe_DIF_; r =  − 0.132, Ar_DIF_; r =  − 0.135).

**Table 4:** Summary statistics and standardized effect size (Hedges’g) for paired t-test between control and experimental plots (controls are set as reference group).

Paired t-test (control versus experimental)				
H_1_: true difference in means is not equal to 0				
Variation metrics	Mean difference	t	*p* value	Hedges’g (95% CI)
Ho	0.001	0.202	0.843	0.049 [− 0.446, 0.546]
uHe	0.000	0.000	1.000	0.000 [0.000, 0.000]
Ar	0.035	0.291	0.775	0.071 [− 0.424, 0.568]
Ho_DIF_	− 0.015	− 1.519	0.151	− 0.371 [− 0.892, 0.137
uHe_DIF_	0.000	0.096	0.924	0.024 [− 0.471, 0.519]
Ar_DIF_	− 0.017	− 0.369	0.717	− 0.090 [− 0.588, 0.405]
σ_bw_	− 0.001	− 0.249	0.806	− 0.061 [− 0.558, 0.434]
Fst	0.000	0.211	0.836	0.052 [− 0.443, 0.548]
G”st	− 0.003	− 0.317	0.756	− 0.077 [− 0.577, 0.417]
r_Gd_	− 0.020	− 1.819	0.090	− 0.444 [− 0.975, 0.071]
r_Dps_	− 0.017	− 1.751	0.102	− 0.427 [− 0.957, 0.086]
r_Lrm_	0.015	1.771	0.098	0.432 [− 0.081, 0.962]

Response variables are: observed heterozygosity (Ho), unbiased expected heterozygosity (uHe), allelic richness (Ar) and their homologous between sides within plots computations (_DIF_), genetic variation within plots (σ_bw_) genetic differentiation metrics (Fst and G”st) and partial mantel r (r_Gd_, r_Dps_, r_Lrm_) as a measure of genetic dissimilarities/similarities within plots.

### Genetic structure and differentiation

The Analysis of Molecular Variance (AMOVA) performed in each plot showed low values of genetic variance among individuals within the same side of the putative barrier (less than 8% in every plot; Φ_IT_ = 0.003–0.077) and extremely small genetic variance between individuals from opposite sides of the putative barrier (less than 3% in every plot; Φ_ST_ =  − 0.006–0.016; Supplementary Table S5). Most of the genetic variance (92.28–99.53%) was explained by differences within individuals (Φ_IS_ = 0.001–0.075). The proportion of variance explained by the presence of the barrier was statistically significant in six out of the 30 (20%) study plots, but significant results were not associated with any particular type of barrier, nor were more prevalent in experimental than in control plots. Plots bisected by highways (H) and combined barriers (CB) tended to have variance ratio values between both plot sides (σ_Between_/σ_Within_) lower than their respective controls, showing a large effect for CB (Supplementary Table S6), whereas plots with conventional roads (R) showed greater values of variance ratio than their controls (Supplementary Fig. S1). However, there were no significant differences between experimental and control treatments (Table 4) and the mixed models showed a lack of significant effect of barrier *Type*. Barrier *Age* and barrier *Width* had again no significant effect on genetic variation, and effect sizes were small in all cases (Supplementary Table S6). The genetic differentiation tests (G-tests) were significant in the six plots with significant AMOVAs, but again not showing any particular pattern with respect to barrier type (data not shown).

**Figure 3:**
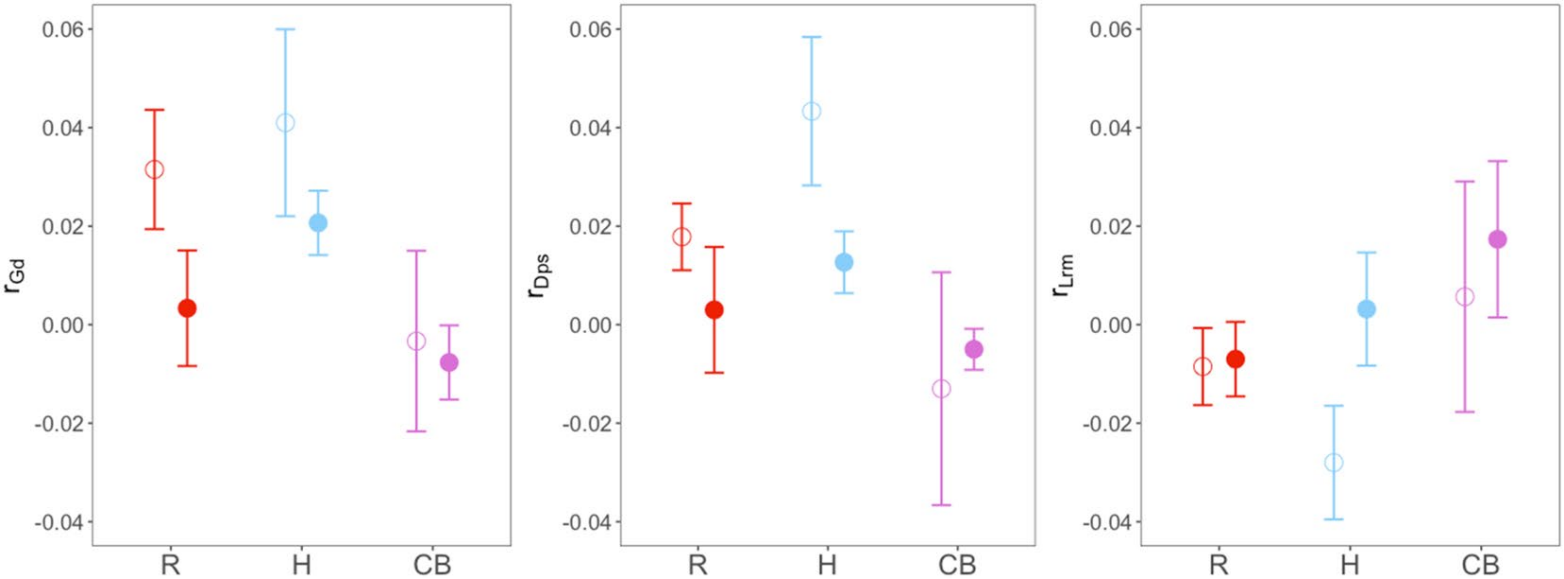
Mean and standard error for Mantel *r* coefficients computed between the matrix of genetic distances (calculated as Gd, Dps and Lrm) and the binary model (designed to separate the same and opposite sides of the barrier), controlling for the effect of Euclidean distances (see text for further explanation). Means are computed for plots belonging to each type of putative barrier (filled dots; R, H, CB) and compared to their control plots (empty dots).

The Mantel’s correlation coefficient computed with the genetic distances (r_Gd_, r_Dps_, see methods section for further explanation and Table 2) showed lower r_Gd_ and r_Dps_ values for roads (R) and highways (H) compared to their controls. As expected, the Mantel’s *r* computed with the relatedness estimator (r_Lrm_) displayed the opposite pattern (Fig. 3). Overall, paired t-test between experimental and control plots did not show significant differences in any Mantel’s statistic (Table 4, and Fig. 3), and effect sizes were all small. Variation of Mantel’s *r* did not depend on the barrier type based on LMMs, although the effect size was large for r_Gd_ in roads (R) and for r_Dps_ and r_Lrm_ in highways (H), showing lower values for r_Gd_ and r_Dps_ and greater values for r_Lrm_ in plots bisected by the infrastructure than in their respective controls (Supplementary Table S7). However, since the 95% CI included zero in both cases, the null hypothesis should not be rejected. *Width* or *Age* showed no statistically significant effect on genetic distance parameters.

**Figure 4:**
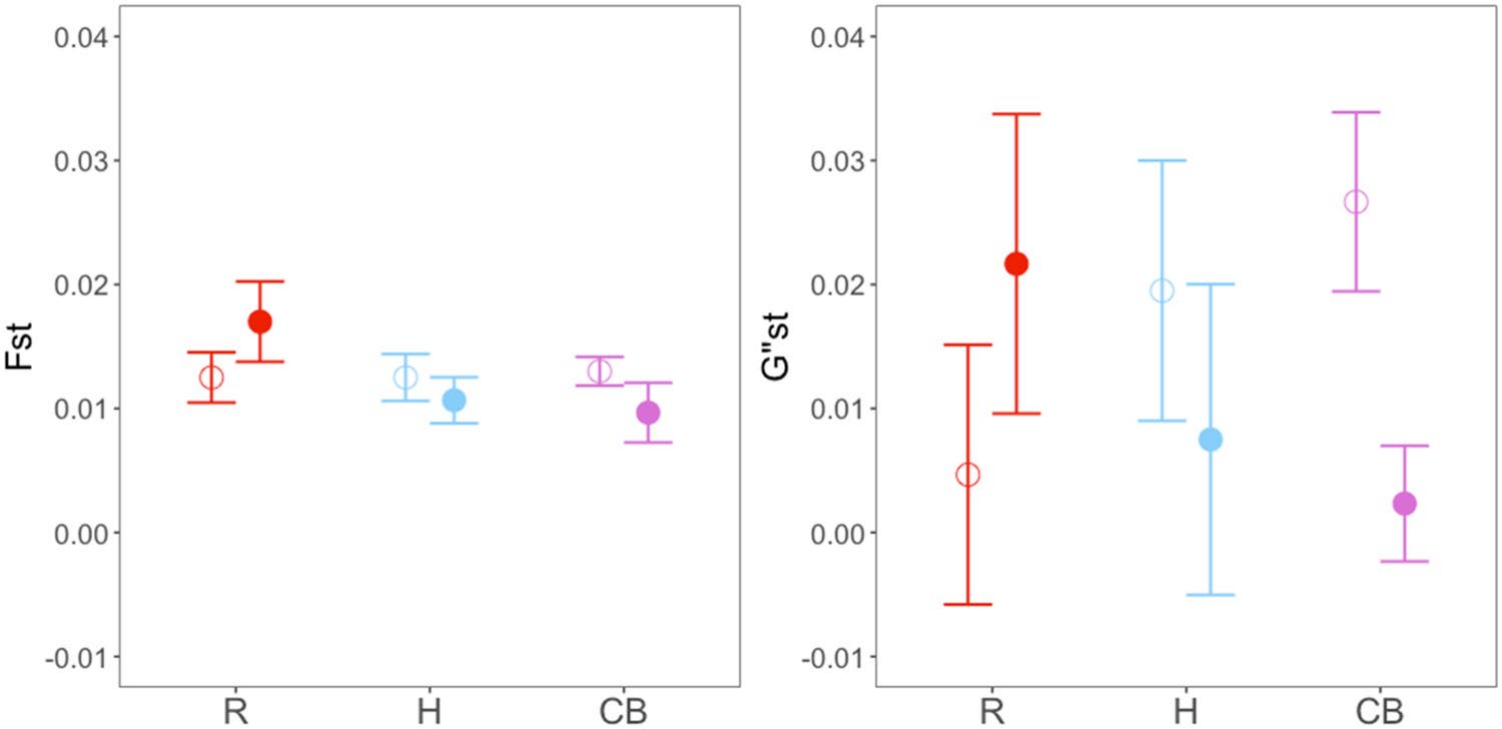
Mean and standard error for genetic differentiation estimators (Fst and G”st) calculated between both sides of the putative barrier within each plot. Means are computed for plots belonging to each type of putative barrier (filled dots; R, H, CB) and compared to their controls (empty dots).

Averaged differentiation indices (Fst and G”st) for experimental and control plots are shown in Fig. 4. Plots crossed by combined barriers (CB) showed lower values of G”st compared to their controls, with large effect size (Supplementary Table S8). Conventional roads (R) showed higher differentiation (Fst and G”st) between barrier sides than their respective controls, but with only medium effect sizes. Nonetheless, paired t-tests did not reveal overall significant differences (Table 4) and neither *Age* nor *Width* appeared to influence these parameters (Supplementary Table S8).

## Discussion

After accounting for the “natural” variation in genetic parameters occurring in our study system through control plots, we could not detect significant evidence to confirm that fragmentation by roads impacts the distribution of genetic variation. We detected a very small, almost null, deviation of genetic parameters from sites without barriers with respect to sites with putative barriers, suggesting either that subpopulations have recently been separated by roads and have not accumulated large differences, that the potential for voles to disperse overcomes the strength of the putative barrier to restrict gene flow, or that vole populations are marginally affected by genetic drift independently of gene flow.

Anthropogenic habitat fragmentation due to road construction is a ubiquitous feature that significantly alters modern landscape structure^59,60^. Generally, by dividing habitats into small fragments, roads act as barriers to gene flow of the isolated of populations. This can leads to adverse genetic effects, especially in species with low dispersal abilities^61^ or in species with low effective population size and thus more vulnerable to loss of genetic variation through drift^62^. A frequent limitation on these studies is that we cannot always possible to directly attribute the observed values of genetic parameters to anthropogenic changes without knowing the situation prior to fragmentation, because such values can result from natural subdivision of populations or may be due to other features present in the landscape, such as the abundance and distribution of habitats or the presence of elements in the landscape that influence individuals’ movement^63^. Unfortunately, we do not have samples available to represent conditions prior to road fragmentation for comparison, so we used a spatially paired design, using comparison between sites with and without paved roads to partially solve this problem^64^.

The genetic effects of fragmentation are expected to become more pronounced through time^65^, and not all methodological approaches are equally effective in detecting a recent barrier. Our studied barriers are relatively recent (time lag from 8 to 61 years-old) and therefore, our ability to detect genetic fragmentation with commonly used genetic indices, such as F_ST_, could be compromised by a short time since fragmentation^34,66^ or by the analytical approach used. However, here we used multiple analytical methods to detect the presence of a linear barrier dividing populations. In addition to population-based approaches that generally requires a longer lag time to detect barriers, we used partial Mantel tests^67^ with individual‐based genetic and geographic distances, which can usually detect the signal of a new barrier within a shorter spatio-temporal scale^34,36^. Additionally, the study model is a short-lived species whose average lifespan is no longer than 4–5 months^68^ and a short-generation time (1 year^69,70^) so the expectation that genetic differences accumulate each generation since fragmentation makes it a suitable species for detecting barriers in the time frame considered^71^. Hence, the lack of evidence for population fragmentation by roads observed in the present work is less due to absence of evidence than evidence of absence. However, our findings do not agree with theoretical and empirical studies that suggest that landscape barriers can have rapid genetic effects that are detectable almost immediately or after a few generations^72–74^, but matches with expectations given the characteristics of the study species, such as its cyclical abundance, the large population size that it reaches in the peak phases and the severe population bottlenecks during the low phase of the cycle, the massive dispersal events associated with population peaks, or the short time lag between these two extremes (typically a few months).

One could argue that the lack of a barrier effect on genetic diversity and structure could be simply due to a large population size, since the amount of change in allele frequencies due to drift decreases as the effective population size increases^51,62^. Therefore, a lack of evidence of road barrier effect could be derived from a marginal effect of drift alone. However, vole population size does not remain high for a long time; the species is well known to suffer “boom-bust” dynamics^75,76^, with a peak every 3–5 generations in which population can fluctuate dramatically from 1000 voles/ha in high-density peaks to 10 voles/ha during population crashes, so it is unlikely that genetic diversity and structure would not be affected by bottleneck-associated drift during low phases.

It is more probable that substantial gene flow occurs across barriers, at least temporally, and that dispersal ability of the species outweighs any influence of the studied features. This is consistent with the lack of strong heterozygote deficiencies, which would be expected if there were an increase in mating events between relatives due to a dispersal decline. Dispersal would increase the geographic scale of genetic structuring, as previously suggested for other cyclic species like Siberian lemmings^77^ and would explain the apparent lack of local genetic structure attributable to roads, matching with previous studies performed on the same species^78^.The analyses of genetic variation in the 30 study sites also revealed an apparent lack of local genetic structure attributable to roads, and the differences in the distribution of variance within and between barrier sides do not seem currently affected by road type. Altogether, these results point to compensating mechanisms, probably related to gene flow and frequent dispersal, that could outweigh the overall impact of roads in this species.

The question remains as to whether the lack of barrier effect observed is due to permanent high dispersal rates over time (i.e. high gene flow across the barrier remains constant from one generation to the next) or if the barrier effect varies through time following changes in Ne. Unfortunately, we do not have temporally replicated data to test this, something that needs to be addressed. However, the well-known cases of barrier effect of roads in similar species and the cyclical nature of the common vole suggests barrier effect varies through time. Roads with similar characteristics to those of this study have been proven effective in reducing the gene flow on a local scale of other mammals similar in size and vagility to common voles, but lacking strong abundance cycles^8^. In cyclic voles, data on dispersal distances are scarce, but the most frequent seem to be short-distance dispersal events carried out mainly by females^79^, which can reach 50–160 m in the case of short-range natal dispersal, but occasionally up to 400 m in a few days^44^. This can lead to significant genetic differentiation even at spatial scales of a few hundred meters^80^. However, common voles are also capable of dispersing on the order of a few kilometers^79^ being these occasional long-distance dispersal events more balanced between genders^80^. The species also shows conspicuous and recurrent episodes of massive dispersal^41,45^ that may genetically homogenize populations. These episodes, although far from clear, are widely understood in the cyclic rodent literature as *presaturation dispersal*^81–83^ and occur under positive growth rates when the patch’s carrying capacity has not yet been reached^81^. Such dispersal events, together with the high effective population size reached during peak phases, may help us to explain the high genetic diversity that common vole maintains in the region. High levels of genetic diversity are well documented for other cyclic small mammals^56,84^, and suggest that population dynamics and dispersal movements play a key role in configuring the genetic diversity of open populations^85^.

In conclusion, our study points out a scenario in which, although there may be periods of relative isolation by roads for a few generations, they regularly alternate with a relatively high panmictic-density phase in which mixing and accumulation of genetic material across barriers occurs, probably due to the cyclic nature of the common vole. These repetitive (cyclic) pulses of gene flow might homogenize populations and erase the possible differences that may have accumulated during the short time elapsed between the “boom” phases. Nevertheless, we cannot conclusively discard that our findings can be explained by other factors independently of gene flow, such as for example a low drift effect and/or the high effective population size during the peak phases. Further research on populations that have been in a low population phase for some time, testing for potential effects of roads over short time scales, would be particularly valuable in that context. We also urge caution in the interpretation of these results to avoid over-emphasizing the low ecological significance of these local barrier effects on populations without larger sample sizes and better temporal sampling coverage, since we detected large effect sizes and trends attributable to roads but which we could not confirm with certainty. A larger number of replicates would give the design the ability to detect smaller differences (higher statistical power) than in the present study, thus reducing the risk for Type-II errors.

Last but not least, the present work underlines the importance of having a robust experimental design and sufficient spatial replication in studies of barrier effects, given the natural spatial variation in genetic parameters. For example, the proportion of genetic variance explained by the putative barrier was statistically significant in the 20% of our study plots, but significant results were not associated with any particular type of barrier, nor were more prevalent in experimental than control plots. This emphasizes the fact that studying the relationship between landscape fragmentation and genetic diversity/structure in single sites can be problematic because the observed genetic parameters are often site-specific and a basis for comparison is lacking, and therefore single-case studies will not allow firm conclusions.

## Methods

### Study area, experimental design and animal collection

We conducted the study in the region of Castilla y León (NW Spain), which includes the Duero River basin, a central agricultural plain (ca. 3.7 million ha; 37.8% of the total area of Castilla y León^86^) characterized by a landscape generally flat with few trees and subject to a moderate-to-high agricultural exploitation^87^. The region combines cities and rural areas, with a well-developed road network that has been expanded and upgraded in recent decades and that includes a wide variety of road types (Fig. 1).

We conducted a field experiment with a matched pairs design using 15 pairs of rectangular study plots (2.5 × 3.0 km, Fig. 1). Each pair consisted of one (experimental) plot longitudinally bisected by a paved transport infrastructure (hereafter: road), leaving approximately 2.5 × 1.5 km at each side of the putative barrier (hereafter: barrier sides), and another (control) plot that was bisected by an unpaved rural track (T). Rural tracks of 4–6 m wide were used as positive controls of the potential barrier effect of major roads since they were assumed to have much less influence on species movement^28,88^ and consequently, on gene flow.

Pairs of experimental and control plots were chosen based on close proximity, with similar environmental and habitat conditions within the agrarian landscape. However, it was not possible to place pairs randomly across the landscape due to the need of: (i) finding plots bisected by a single road, with no other nearby infrastructures, (ii) having dirt tracks that crossed control plots in similar way to roads in experimental plots, and (iii) ease of simultaneous access to multiple sites during mammal trapping. We also avoided sites with any obvious signs of disturbance such as burning, human settlements and other constructions, that could confound analysis of the effects of roads on genetic differentiation. Therefore, we located sampling sites on flat terrain and avoiding any field with streams that were parallel to the road, thus avoiding possible additional barriers to common vole gene flow^89,90^.

The two plots in each pair (treatment and its control) were spaced 2–4 km apart, and each set of pairs was separated by 6–26 km to promote independence. We considered three treatments (types of road) in the experimental design, each one having intrinsic features (Supplementary Table S9): conventional 2 lane-roads (R) that represented the oldest and narrowest paved transport infrastructures in the region, 4-lane highways (H) with intermediate ages and sizes, and combined barriers (CB), where R and H run parallel and proximate (Fig. 1), that represented the widest and most recent infrastructures. Specific road features like width (considered as the distance between the outermost edges—including paved lanes plus gravel shoulders/drainage systems—of the infrastructure) were obtained using Google Earth and data from the Spanish Ministry of Development, whereas the historical imagery of Google Earth and the stereoscopic aerial images available from the American Flight B series (AMS 1956–1957) to present, allowed us to calculate the year of construction (data provided by Confederación Hidrográfica del Duero).

Voles were live-trapped in 30 study plots from late April to the end of July 2017, the year following a population peak phase in the region^91^. We established Sherman traps (H.B. Sherman Traps Inc. Florida, USA) in 26–96 capture points per study plot, with 10 traps per point, deployed through the entire plot (260–960 traps per study plot). We tried to ensure that trap locations encompassed the diversity of habitats (e.g., crop fields, fallows, field margins, boundaries of roads and rural tracks, ditches), but > 500 m from plot boundaries to reduce potential edge effects^92^. Traps were open for 24 h, or until captures reached a minimum of at least 20 individuals per infrastructure side, which usually took no more than 48 h. However, we were not always able to reach the desired number of 20 samples per plot side (see Table 3). From each individual, we took 2 mm tissue biopsies from the ear and preserved them in 96% ethanol. All animals were released back to their exact point of capture. Precise locations of each trapping site were quantified using a hand-held GPS (± 4 m error).

All procedures were approved by the University of Castilla-La Mancha's Committee on Ethics and Animal Experimentation (CEEA, UCLM, Spain; reference number PR20170201), were within the guidelines of the European and Spanish and policy for animal protection and experimentation and in compliance with the ARRIVE guidelines for how to report animal experiments.

### DNA extraction and microsatellite genotyping

Total genomic DNA was extracted from tissue samples using a standard ammonium acetate protocol^93^, and diluted to a working concentration of 25 ng/μL. 9 microsatellite loci isolated in common vole^94,95^ were amplified in a total volume of 10 μL containing 1 × QIAGEN Multiplex PCR Master Mix, 0.2 μM of each primer (forward and reverse) and 20–50 ng of DNA. The 9 primer pairs were divided into two multiplexes using four dyes (FAM, PET, NED, VIC): Ma09, Mar012, Ma54, Ma66, (Multiplex 1); MM6, Mar063, Mar003, Mar016, Mar102 (Multiplex 2). Electrophoresis of PCR products was performed by Universidad Complutense de Madrid, Unidad de Genómica, Spain (https://www.ucm.es/gyp/genomica). Electropherograms were scored using Geneious 10.2.3 (https://www.geneious.com) and approximately 10% of all genotypes were re-amplified and re-scored to estimate the genotyping error rate.

Prior to statistical analysis, microsatellite error rate was calculated to check for allelic dropout and false alleles using Pedant v1.0^96^. Hardy Weinberg equilibrium and Linkage disequilibrium were checked across all loci in the R package ‘pegas’^97^. We checked for the presence of null alleles following Brookfield^98^ implemented in the R package ‘PopGenReport v3.0.4′^99^. We used COLONY 2.0^100^ to identify and discard full and half siblings at the same sampling point from the dataset, which may indicate the presence of litters and bias the allele frequencies at a given spatial location. COLONY was run under a non-restrictive criterion considering a polygynandrous scenario (both male and female potentially polygamous). Analyses were performed according with the full-likelihood method with high precision, three runs of medium length and without priors. Since COLONY provides the uncertainty estimates (in probabilities) of a particular substructure, we assess, for each of our sampling points, the probability that the captured individuals share both (full siblings) or one parent (half-siblings) using a threshold of substructure probability *p* ≥ 0.95. Then, we randomly discard all but one full sibling from each sampling location. Siblings sampled at different sampling points were retained for analysis.

### Genetic diversity

We estimated genetic diversity through ‘diveRsity*’* v1.1.9^101^ R package. We first calculated observed (Ho) and expected (He) heterozygosity, unbiased expected heterozygosity (uHe), allelic richness (Ar) and inbreeding coefficient (F_IS_). We used ‘GENHET’ v3.1^102^ R package to calculate five measures of genetic diversity at the individual level, as described in Coulon^102^: the proportion of heterozygous loci (PHt), standardized heterozygosity based on the mean observed heterozygosity (Hs_obs), standardized heterozygosity based on the mean expected heterozygosity (Hs_exp^103^), internal relatedness (IR^104^), and homozygosity by locus (HL^105^). All metrics of genetic diversity were calculated (i) as a mean value per plot, using all samples collected in each plot, and (ii) as a mean value in each side of the putative barrier within each plot (arbitrarily coded as A and B). We then computed an absolute difference across the barrier to obtain a single value per plot (Ho_DIF_, He_DIF_, uHe_DIF_, Ar_DIF_, Fis_DIF_, PHt_DIF_, Hs_obs_DIF_, Hs_exp_DIF_, IR_DIF_, HL_DIF_). To verify that the observed values for these estimates (differences in diversity) were not just a consequence of sample size, we computed a Pearson’s correlation coefficient between our diversity parameters and the sample size per plot. In a possible scenario of subtle isolation due to roads, we would expect that although there were no differences in the overall diversity between an experimental plot and its control, we found differences in the way that diversity was distributed in relation to the position of the barrier (and absence of differences among sides in the control plot).

### Genetic structure and differentiation

Based on AMOVA and Phi-statistics, genetic variation between the two roadsides was estimated for all plots using three hierarchical levels: roadside, sampling site (plot) and individual. For the assessment of potential barriers, individuals where assigned to their respective roadside. AMOVA was performed with 999 permutations to assess significance in ‘poppr’^106^ R package. Regardless of the outcome of the AMOVA tests computed in each plot, our aim was to identify an overall difference in the variance explained by the putative barrier between the experimental plots and their paired controls. We then computed the ratio of the genetic variance between different sides of the barrier to the variance on the same side of the barrier (σ_bw_ = σ_Between_/σ_Within_). This parameter was used as a response variable in subsequent analysis (see below), which was expected to be greater in the presence of a barrier effect. GENALEX v6.5^107^ was also used to assess pairwise genetic differentiation (F_ST_) between groups of individuals at each side of the putative barrier within each plot, therefore testing genetic structuring by the barrier, and statistical significance based on 999 permutations. Recent analyses suggest that this standard measure of differentiation may be poorly suited for data sets in which allelic diversity is high^108–110^. Given the high variability of the markers used here, we also computed other measures of differentiation: G_ST_^111^_,_ G”_ST_^112^, G’_ST_H^108^ and D^109^ following the recommendations and formulae of Meirmans and Hedrick^110^.

Because some authors^113,114^ have suggested that metrics based on genetic differentiation among groups of individuals may have low power to detect isolation, we also computed individual-based genetic distances within each plot. Pairwise individual genetic distances were estimated using the proportion of shared alleles (Dps^113^) and codominant genotypic distance (Gd^115^). For Dps, we subtracted values from one to obtain a distance measure and make it comparable with Gd. We additionally computed three relatedness indicators per plot: Qgm^116^, Ri^117^ and Lrm^118^ that measure genetic similarity in terms of probabilities of identity-by-descent including the overall population allele frequencies as a parameter (in our case, the average allele frequencies calculated over the 30 plots). We multiplied Lrm by two to make it comparable with the other relatedness estimators, but we did not subtract values in this case since negative values are informative for these metrics. Gd and relatedness estimators were calculated in GENALEX and Dps in “adegenet” v2.1.1^119^ R package.

As we were aware of the potential spatial autocorrelation of individuals in our plots and that spatial dependence can interfere with our ability to make statistical inferences^120^, we also calculated pairwise Euclidean geographic distances among individuals. We then used genetic, geographic, and a third “barrier” matrix to compute a partial Mantel test per plot. The elements of our barrier matrix were binary (i.e., 0 between samples from the same side of the putative barrier, 1 between samples separated by the putative barrier). Under the null hypothesis (H_0_), one would expect that the genetic distances among samples are unrelated to the corresponding treatment distances (while controlling for corresponding geographic distances). Under the alternative hypothesis (H_1_) we expected to find significant correlation between genetic and treatment distances. Therefore, Mantel *r* coefficient was expected to be greater in the test carried out with the experimental plots than with the control plots.

### Statistical analysis

Due to the large number of metrics employed in the study, we retained only those which provided non-redundant information based on a correlation matrix with a Pearson’s coefficient threshold < 0.80^121^ to be used as response variables. These included Fst and G”st as differentiation metrics, σbw as a measure of genetic variation, Ho, uHe, Ar and their homologous computations of differences between plot sides (Ho_DIF_, uHe_DIF_, Ar_DIF_) as genetic diversity estimators, and partial Mantel r (r_Gd,_ r_Dps_, r_Lrm_). A detailed explanation of each estimator is given in Table 2.

First, we performed paired t-tests between experimental and their respective controls for each group of metrics to assess a *Treatment* effect. Then, we constructed Linear Mixed-effect Models (LMMs) to analyze variation in genetic metrics according to the type of putative barriers, including barrier *Type* as a fixed factor with the three treatments (R, H, CB) and the control (T) as factor levels. Because of the nature of our sampling design, each experimental plot and its paired control were coded with the same identification number, which was included in the models as a random factor (*GroupID*) to make appropriate paired comparisons. We also conducted separate analyses to examine the effect of road *Width* and *Age* on genetic metrics. We restricted this analysis to highways, as they had larger contrast relative to the control plots than roads, which had all the same age, and lacked infrastructures with different age and width (CB), which potentially complicates interpretation. Therefore, we performed simple linear regressions after verifying that there was no significant correlation between *Age* and *Width* of the barrier for this data subset (r = 0.043, *p* = 0.895). Since this is a matched pair study we used the differences in genetic parameters between each experimental plot and its paired control plot as response variable in the linear regressions.

Due to the low sample size in both sets of models we cannot rule out the possibility of insufficient statistical power. Therefore, we estimated the effect size by computing Hedges’s g^122^, corrected for small sample sizes, with ‘bootES’^123^ R package, and used the threshold provided in Ref.^124^ to assess the magnitude of the effect in paired t-tests and LMMs, i.e. *|g|*< 0.2: negligible; 0.2 ≤*|g|*≤ 0.5: small; 0.5 ≤*|g|*≤ 0.8: medium; *|g|*> 0.8: large. We bootstrapped 999 times on effect sizes to get the 95% confidence intervals, which can inform inferences about the replicability and generalizability of the results. In the same way, we computed Cohen’s f^2^ for simple regression models as described in Ref.^125^ for the highways (H) subset: 0.02 ≤|*f*^2^*|*< 0.15: small; 0.15 ≤|*f*^2^*|*< 0.35: medium; |*f*^2^*|*≥ 0.35: large.

## Supplementary Information

Supplementary Information.

## Data Availability

The datasets generated during and/or analyzed during the current study are available from the corresponding author on reasonable request.
