# Supplementary material for: Lack of detectable genetic isolation in the cyclic rodent Microtus arvalis despite large landscape fragmentation owing to transportation infrastructures
Source: Sci Rep. 2021 Jun 15;11:12534. doi: 10.1038/s41598-021-91824-w (PMC8206325; doi:10.1038/s41598-021-91824-w)
Supplement: Supplementary file 1 — Supplementary Information. [file 41598_2021_91824_MOESM1_ESM.docx]

**Supplementary material**

**Lack of detectable genetic isolation in the cyclic rodent *Microtus arvalis* despite large landscape fragmentation owing to transportation infrastructures**

Julio C. Dominguez^1^*, María Calero-Riestra^1^, Pedro P. Olea^2,3^, Juan E. Malo^2,3^, Christopher P. Burridge^4^, Kirstin Proft^4^, Sonia Illanas^1^, Javier Viñuela^1^, Jesús T. García^1^.

^1^IREC, Instituto de Investigación en Recursos Cinegéticos (CSIC-UCLM-JCCM), Ronda de Toledo 12 13071 Ciudad Real, Spain.

^2^Terrestrial Ecology Group (TEG-UAM), Departamento de Ecología, Facultad de Ciencias, Universidad Autónoma de Madrid, C/Darwin 2, 28049 Madrid, Spain.

^3^Centro de Investigación en Biodiversidad y Cambio Global (CIBC-UAM), Universidad Autónoma de Madrid. C. Darwin 2, 28049 Madrid, Spain.

^4^Discipline of Biological Sciences, University of Tasmania, Private Bag 55, Hobart, TAS, 7001, Australia.

*Corresponding author. E-mail address: jcesardv@gmail.com

**Supplementary Table S1.** Adjusted p-values for the HWE test using MCMC with 9999 permutations. Significant loci are indicated in red.

| **Plot** | **Ma09** | **Mar102** | **Mar012** | **Mar003** | **Mar063** | **Mar016** | **MM6** | **Ma54** | **Ma66** |
| --- | --- | --- | --- | --- | --- | --- | --- | --- | --- |
| CB1 | 0.052 | 0.080 | 0.940 | 0.558 | 0.038 | 0.029 | 0.830 | 0.762 | 0.173 |
| CB1c | 0.054 | 0.076 | 0.942 | 0.559 | 0.036 | 0.031 | 0.831 | 0.766 | 0.169 |
| CB2 | 0.050 | 0.082 | 0.939 | 0.559 | 0.039 | 0.029 | 0.827 | 0.769 | 0.173 |
| CB2c | 0.051 | 0.079 | 0.938 | 0.552 | 0.039 | 0.028 | 0.820 | 0.769 | 0.172 |
| CB3 | 0.029 | 0.745 | 0.056 | 0.112 | 0.328 | 0.494 | 0.390 | 0.026 | 0.085 |
| CB3c | 0.579 | 0.538 | 0.266 | 0.148 | 0.410 | 0.158 | 0.142 | 0.720 | 0.217 |
| H1 | 0.931 | 0.420 | 0.473 | 0.935 | 0.077 | 0.961 | 0.363 | 0.023 | 0.979 |
| H1c | 0.306 | 0.094 | 0.160 | 0.489 | 0.198 | 0.139 | 0.471 | 0.566 | 0.896 |
| H2 | 0.115 | 0.226 | 0.271 | 0.341 | 0.173 | 0.552 | 0.718 | 0.028 | 0.867 |
| H2c | 0.714 | 0.176 | 0.566 | 0.422 | 0.212 | 0.771 | 0.536 | 0.208 | 0.588 |
| H3 | 0.250 | 0.230 | 0.272 | 0.232 | 0.024 | 0.094 | 0.724 | 0.057 | 0.297 |
| H3c | 0.436 | 0.958 | 0.384 | 0.482 | 0.961 | 0.688 | 0.007 | 0.092 | 0.373 |
| H4 | 0.836 | 0.274 | 0.025 | 0.688 | 0.059 | 0.981 | 0.034 | 0.405 | 0.188 |
| H4c | 0.726 | 0.549 | 0.076 | 0.010 | 0.019 | 0.762 | 0.295 | 0.311 | 0.482 |
| H5 | 0.496 | 0.794 | 0.036 | 0.941 | 0.622 | 0.354 | 0.433 | 0.649 | 0.927 |
| H5c | 0.920 | 0.405 | 0.006 | 0.909 | 0.028 | 0.238 | 0.226 | 0.322 | 0.396 |
| H6 | 0.062 | 0.878 | 0.313 | 0.612 | 1.000 | 0.926 | 0.355 | 0.194 | 0.598 |
| H6c | 0.564 | 0.685 | 0.000 | 0.213 | 0.195 | 0.034 | 0.221 | 0.821 | 0.229 |
| R1 | 0.292 | 0.397 | 0.324 | 0.832 | 0.157 | 0.663 | 0.373 | 0.139 | 0.455 |
| R1c | 0.579 | 0.706 | 0.451 | 0.372 | 1.000 | 0.452 | 0.322 | 0.055 | 0.812 |
| R2 | 0.554 | 0.467 | 0.769 | 0.726 | 0.165 | 0.890 | 0.849 | 0.334 | 0.849 |
| R2c | 0.750 | 0.104 | 0.420 | 0.986 | 0.830 | 0.523 | 0.759 | 0.167 | 0.633 |
| R3 | 0.697 | 0.859 | 0.235 | 0.527 | 0.183 | 0.537 | 0.749 | 0.006 | 0.139 |
| R3c | 0.936 | 0.832 | 0.142 | 0.050 | 1.000 | 0.266 | 0.896 | 0.685 | 0.008 |
| R4 | 0.695 | 0.093 | 0.465 | 0.455 | 0.069 | 0.964 | 0.484 | 0.514 | 0.535 |
| R4c | 0.058 | 0.710 | 0.097 | 0.557 | 0.040 | 0.041 | 0.479 | 0.753 | 0.135 |
| R5 | 0.067 | 0.262 | 0.620 | 0.058 | 0.388 | 0.746 | 0.063 | 0.927 | 0.104 |
| R5c | 0.070 | 0.151 | 0.036 | 0.483 | 0.573 | 0.437 | 0.020 | 0.448 | 0.857 |
| R6 | 0.719 | 0.250 | 0.724 | 0.997 | 1.000 | 0.613 | 0.829 | 0.319 | 0.975 |
| R6c | 0.252 | 0.641 | 0.119 | 0.200 | 1.000 | 0.881 | 0.702 | 0.779 | 0.808 |

**Supplementary Table S2.** Loci with possible null alleles identified by PopGenReport. Null allele frequencies are estimated within each plot according to Brookfield’s method. (Brookfield et al. 1996).

| **Plot** | **Ma09** | **Mar102** | **Mar012** | **Mar003** | **Mar063** | **Mar016** | **MM6** | **Ma54** | **Ma66** |
| --- | --- | --- | --- | --- | --- | --- | --- | --- | --- |
| CB1 | 0.02 | 0.03 | -0.01 | 0.00 | -0.01 | -0.02 | -0.01 | 0.01 | 0.01 |
| CB1c | 0.02 | -0.03 | -0.01 | 0.07 | 0.00 | -0.01 | -0.01 | -0.01 | 0.00 |
| CB2 | 0.00 | -0.01 | 0.04 | -0.05 | 0.02 | -0.01 | 0.04 | 0.01 | 0.01 |
| CB2c | -0.01 | 0.01 | 0.01 | -0.01 | -0.01 | -0.01 | 0.00 | 0.05 | -0.03 |
| CB3 | 0.01 | -0.03 | 0.02 | 0.00 | 0.04 | -0.06 | 0.04 | 0.08 | 0.05 |
| CB3c | -0.01 | -0.02 | 0.06 | 0.02 | 0.02 | 0.01 | 0.03 | 0.01 | 0.03 |
| H1 | -0.04 | 0.02 | 0.04 | -0.02 | 0.03 | -0.02 | -0.02 | 0.09 | -0.03 |
| H1c | -0.01 | 0.01 | -0.02 | 0.00 | 0.03 | 0.04 | 0.00 | 0.04 | -0.04 |
| H2 | 0.01 | 0.01 | 0.02 | 0.02 | 0.05 | 0.01 | -0.01 | 0.00 | -0.03 |
| H2c | -0.07 | 0.04 | -0.01 | 0.01 | -0.01 | -0.01 | 0.05 | 0.00 | 0.00 |
| H3 | 0.01 | 0.03 | 0.00 | 0.03 | 0.04 | 0.06 | -0.01 | 0.02 | -0.02 |
| H3c | 0.02 | 0.01 | -0.01 | -0.01 | -0.03 | 0.01 | 0.05 | 0.05 | 0.03 |
| H4 | -0.02 | 0.00 | 0.06 | 0.01 | 0.03 | -0.03 | 0.01 | 0.06 | 0.02 |
| H4c | 0.02 | 0.05 | 0.05 | 0.05 | 0.05 | -0.01 | 0.01 | -0.06 | -0.02 |
| H5 | -0.02 | -0.03 | 0.08 | 0.02 | 0.02 | -0.01 | -0.05 | 0.01 | 0.01 |
| H5c | -0.04 | 0.00 | 0.06 | -0.03 | 0.03 | 0.01 | 0.02 | 0.01 | -0.03 |
| H6 | -0.03 | -0.01 | 0.05 | -0.04 | -0.02 | 0.01 | 0.02 | 0.02 | 0.03 |
| H6c | 0.01 | 0.00 | 0.14 | 0.01 | 0.01 | 0.03 | 0.03 | -0.02 | 0.05 |
| R1 | 0.00 | -0.01 | 0.00 | -0.02 | 0.00 | 0.01 | 0.01 | -0.03 | 0.01 |
| R1c | 0.03 | -0.01 | 0.00 | 0.01 | -0.02 | -0.02 | -0.04 | 0.06 | 0.00 |
| R2 | -0.02 | -0.05 | 0.01 | 0.00 | 0.02 | 0.01 | -0.01 | 0.02 | -0.01 |
| R2c | -0.02 | -0.04 | 0.04 | 0.01 | -0.05 | -0.02 | 0.01 | 0.06 | -0.01 |
| R3 | 0.00 | 0.03 | 0.01 | -0.02 | 0.04 | 0.04 | 0.01 | 0.08 | 0.00 |
| R3c | -0.03 | 0.00 | 0.05 | 0.04 | -0.02 | 0.05 | 0.02 | -0.04 | -0.01 |
| R4 | -0.02 | 0.00 | 0.03 | 0.00 | 0.01 | -0.05 | -0.03 | 0.05 | 0.03 |
| R4c | -0.01 | -0.02 | 0.02 | 0.00 | 0.08 | 0.04 | 0.03 | -0.01 | 0.02 |
| R5 | -0.02 | 0.01 | 0.00 | 0.02 | 0.03 | 0.00 | 0.03 | 0.02 | 0.01 |
| R5c | -0.01 | 0.02 | 0.01 | 0.02 | -0.03 | 0.00 | -0.01 | 0.00 | -0.02 |
| R6 | -0.02 | 0.01 | -0.03 | -0.04 | -0.05 | -0.07 | -0.03 | 0.01 | -0.01 |
| R6c | 0.02 | -0.02 | 0.03 | 0.01 | -0.01 | -0.04 | 0.03 | 0.03 | 0.03 |

**Supplementary Table S3.** Results for linear mixed model (y ~ Type + 1|GroupID) (A) and simple regressions using *Age* (B) and *Width* (C) as predictors. Response variables are: observed heterozygosity (Ho), unbiased expected heterozygosity (uHe) and allelic richness (Ar). For *Age* and *Width* models, response variables were computed as differences (Δ) between experimental and their associated controls for 4-lane highways, exclusively (see text for further details). GroupID: a unique identifier for each pair of experimental/control plots. Intercept in A represents the level taken as reference for contrasts (unpaved rural tracks, T).

|  | **Ho** | | | | **uHe** | | | | **Ar** | | | |
| --- | --- | --- | --- | --- | --- | --- | --- | --- | --- | --- | --- | --- |
| **A** | F | df | p | f^2^ | F | df | p | f^2^ | F | df | p | f^2^ |
| Barrier Type | 0.179 | 3.000 | 0.909 | 0.018 | 0.620 | 3.000 | 0.613 | 0.046 | 0.384 | 3.000 | 0.766 | 0.020 |
|  | Estimate | SE | p | g | Estimate | SE | p | g | Estimate | SE | p | g |
| (Intercept) | 0.788 | 0.006 | <0.001 |  | 0.807 | 0.004 | <0.001 |  | 9.908 | 0.170 | <0.001 |  |
| R | 0.002 | 0.010 | 0.850 | 0.161 | -0.003 | 0.006 | 0.611 | 0.000 | -0.118 | 0.194 | 0.552 | -0.138 |
| H | -0.003 | 0.010 | 0.764 | 0.000 | -0.002 | 0.006 | 0.738 | -0.094 | 0.114 | 0.194 | 0.566 | 0.208 |
| CB | 0.009 | 0.014 | 0.519 | -0.125 | 0.010 | 0.008 | 0.231 | 0.165 | 0.185 | 0.270 | 0.504 | 0.000 |
| **B** | Estimate | SE | p | f^2^ | Estimate | SE | p | f^2^ | Estimate | SE | p | f^2^ |
| (Intercept) | -0.026 | 0.013 | 0.108 |  | -0.002 | 0.011 | 0.870 |  | 1.491 | 0.666 | 0.089 |  |
| Age | 0.001 | 0.001 | 0.096 | **1.176** | 0.000 | 0.001 | 0.982 | 0.000 | -0.065 | 0.031 | 0.105 | **1.094** |
| **C** | Estimate | SE | p | f^2^ | Estimate | SE | p | f^2^ | Estimate | SE | p | f^2^ |
| (Intercept) | -0.002 | 0.030 | 0.953 |  | -0.017 | 0.016 | 0.332 |  | 0.099 | 1.542 | 0.952 |  |
| Width | 0.000 | 0.001 | 0.952 | 0.001 | 0.000 | 0.000 | 0.367 | 0.259 | 0.001 | 0.029 | 0.967 | 0.000 |
| F=F value, df=degrees of freedom, p=p-value at 95% CI, SE=standard error, g=Hedges’s g, f^2^=Cohen’s f^2^ | | | | | | | | | | | | |

**Supplementary Table S4.** Results for linear mixed model (y ~ Type + 1|GroupID) (A) and simple regressions using *Age* (B) and *Width* (C) as predictors). Response variables: observed heterozygosity (Ho), unbiased expected heterozygosity (uHe) and allelic richness (Ar) were computed as absolute differences between barrier sides (_DIF_) and logarithmically transformed to fit with normality assumptions. For *Age* and *Width* models, response variables were computed as differences (Δ) between experimental and their associated controls for 4-lane highways, exclusively (see text for further details). GroupID: a unique identifier for each pair of experimental/control plots. Intercept in A represents the level taken as reference for contrasts (unpaved rural tracks, T).

|  | **Ho_DIF_** | | | | **uHe_DIF_** | | | | **Ar_DIF_** | | | |
| --- | --- | --- | --- | --- | --- | --- | --- | --- | --- | --- | --- | --- |
| **A** | F | df | p | f^2^ | F | df | p | f^2^ | F | df | p | f^2^ |
| Barrier Type | 1.043 | 3.000 | 0.394 | 0.111 | 1.425 | 3.000 | 0.268 | 0.151 | 0.357 | 3.000 | 0.785 | 0.041 |
|  | Estimate | SE | p | g | Estimate | SE | p | g | Estimate | SE | p | g |
| (Intercept) | 0.033 | 0.007 | 0.000 |  | 0.013 | 0.002 | 0.000 |  | 0.378 | 0.042 | 0.000 |  |
| R | -0.019 | 0.012 | 0.129 | **-0.851** | -0.005 | 0.004 | 0.166 | -0.523 | 0.029 | 0.077 | 0.707 | **0.878** |
| H | -0.010 | 0.012 | 0.421 | -0.085 | 0.005 | 0.004 | 0.185 | 0.534 | -0.069 | 0.077 | 0.378 | **-1.056** |
| CB | -0.019 | 0.016 | 0.255 | -0.381 | 0.002 | 0.005 | 0.721 | 0.000 | -0.025 | 0.101 | 0.809 | -0.132 |
| **B** | Estimate | SE | p | f^2^ | Estimate | SE | p | f^2^ | Estimate | SE | p | f^2^ |
| (Intercept) | -0.050 | 0.056 | 0.420 |  | 0.003 | 0.012 | 0.788 |  | 0.067 | 0.092 | 0.508 |  |
| Age | 0.002 | 0.003 | 0.420 | 0.201 | 0.000 | 0.001 | 0.866 | 0.008 | -0.009 | 0.004 | 0.115 | **1.008** |
| **C** | Estimate | SE | p | f^2^ | Estimate | SE | p | f^2^ | Estimate | SE | p | f^2^ |
| (Intercept) | -0.124 | 0.075 | 0.174 |  | 0.006 | 0.019 | 0.770 |  | -0.080 | 0.208 | 0.721 |  |
| Width | 0.002 | 0.001 | 0.174 | **0.680** | 0.000 | 0.000 | 0.979 | 0.000 | -0.001 | 0.004 | 0.894 | 0.005 |
| F=F value, df=degrees of freedom, p=p-value at 95% CI, SE=standard error, g=Hedges’s g, f^2^=Cohen’s f^2^ | | | | | | | | | | | | |

**Supplementary Table S5.** Analysis of molecular variance (AMOVA) within each plot. Phi-statistics represent: genetic variation between individuals of different sides of the barrier (Φ_ST_), individuals on same side of the barrier (Φ_IS_) and variation within individuals (Φ_IT_**)**. Percentage of total variance is shown in brackets.

| **Plot** | **Φ_ST_ (%)** | **Φ_IT_ (%)** | **Φ_IS_ (%)** |
| --- | --- | --- | --- |
| CB1 | 0.001 (0.12) | 0.010 (0.84) | 0.008 (99.04) |
| CB1c | 0.007 (0.69) | 0.018 (1.06) | 0.011 (98.25) |
| CB2 | 0.001 (0.11) | 0.023 (2.20) | 0.022 (97.69) |
| CB2c | 0.002 (0.25) | 0.012 (0.93) | 0.009 (98.82) |
| CB3 | -0.001 (-0.07) | 0.043* (4.34) | 0.043* (95.74) |
| CB3c | 0.005 (0.53) | 0.044* (3.82) | 0.038* (95.65) |
| H1 | 0.013* (1.30) | 0.025 (1.17) | 0.012 (97.53) |
| H1c | 0.010* (0.97) | 0.021 (1.11) | 0.011 (97.92) |
| H2 | 0.004 (0.38) | 0.026 (2.24) | 0.023 (97.38) |
| H2c | 0.011 (1.05) | 0.020 (0.98) | 0.010 (97.97) |
| H3 | -0.002 (-0.17) | 0.040* (4.17) | 0.042* (96.01) |
| H3c | 0.000 (-0.01) | 0.035* (3.55) | 0.035* (96.46) |
| H4 | -0.001 (-0.09) | 0.039* (3.96) | 0.040* (96.12) |
| H4c | 0.002 (0.20) | 0.030 (2.85) | 0.029 (96.96) |
| H5 | 0.001 (0.12) | 0.012 (1.07) | 0.011 (98.80) |
| H5c | 0.004 (0.41) | 0.014 (0.99) | 0.010 (98.60) |
| H6 | -0.005 (-0.48) | 0.014 (1.89) | 0.019 (98.58) |
| H6c | 0.002 (0.21) | 0.077* (7.52) | 0.075* (92.28) |
| R1 | -0.002 (-0.21) | 0.003 (0.52) | 0.005 (99.69) |
| R1c | 0.000 (0.01) | 0.007 (0.74) | 0.007 (99.26) |
| R2 | 0.016* (1.56) | 0.030 (1.46) | 0.015 (96.97) |
| R2c | -0.002 (-0.23) | 0.020 (2.21) | 0.022 (98.03) |
| R3 | -0.002 (-0.17) | 0.050* (5.16) | 0.052* (95.00) |
| R3c | 0.011* (1.06) | 0.029 (1.89) | 0.019 (97.06) |
| R4 | 0.005 (0.52) | 0.023 (1.74) | 0.017 (97.74) |
| R4c | -0.006 (-0.58) | 0.052* (5.75) | 0.057* (94.83) |
| R5 | 0.010* (1.04) | 0.033 (2.22) | 0.022* (96.74) |
| R5c | 0.004* (0.37) | 0.005 (0.09) | 0.001 (99.53) |
| R6 | 0.002 (0.25) | 0.004 (0.51) | 0.001 (99.24) |
| R6c | -0.001 (-0.12) | 0.031 (3.19) | 0.032 (96.92) |

**Supplementary Table S6.** Results for linear mixed model (y ~ Type + 1|GroupID) (A) and simple regressions using *Age* (B) and *Width* (C) as predictors. Response variable (σ_bw_) is a ratio computed dividing σ_between_ (percentage of variance distributed between populations -individuals from opposite sides of the barrier-) by σ_within_ (percentage of variance distributed within populations, -i.e., pooled samples from same side of the barrier and considered as populations-). For *Age* and *Width* models, response variables were computed as differences (Δ) between experimental and their associated controls for 4-lane highways, exclusively (see text for further details). GroupID: a unique identifier for each pair of experimental/control plots. Intercept in A represents the level taken as reference for contrasts (unpaved rural tracks, T).

| **σ_bw_** | | | | |
| --- | --- | --- | --- | --- |
| **A** | F | df | p | f^2^ |
| Type | 0.635 | 3.000 | 0.601 | 0.076 |
|  | Estimate | SE | p | g |
| (Intercept) | 0.006 | 0.003 | 0.029 |  |
| R | 0.004 | 0.005 | 0.459 | 0.591 |
| H | -0.003 | 0.005 | 0.518 | -0.526 |
| CB | -0.006 | 0.007 | 0.379 | **-1.004** |
| **B** | Estimate | SE | p | f^2^ |
| (Intercept) | -0.003 | 0.011 | 0.829 |  |
| Age | 0.000 | 0.001 | 0.751 | 0.029 |
| **C** | Estimate | SE | p | f^2^ |
| (Intercept) | -0.010 | 0.017 | 0.603 |  |
| Width | 0.000 | 0.000 | 0.833 | 0.013 |
| F=F value, df=degrees of freedom, p=p-value at 95% CI, SE=standard error, g=Hedges’s g, f^2^=Cohen’s f^2^ | | | | |

**Supplementary Table S7.** Results for linear mixed model (y ~ Type + 1|GroupID) (A) and simple regressions using *Age* (B) and *Width* (C) as predictors. Response variables are partial Mantel r computed with codominant genotypic distance (Gd), proportion of shared alleles (Dps) and relatedness estimator (Lrm). For *Age* and *Width* models, response variables were computed as differences (Δ) between experimental and their associated controls for 4-lane highways, exclusively (see text for further details). GroupID: a unique identifier for each pair of experimental/control plots. Intercept in A represents the level taken as reference for contrasts (unpaved rural tracks, T). Large effect sizes are highlighted in bold.

| **A** | **r_Gd_** | | | | **r_Dps_** | | | | **r_Lrm_** | | | |
| --- | --- | --- | --- | --- | --- | --- | --- | --- | --- | --- | --- | --- |
|  | F | df | p | f^2^ | F | df | p | f^2^ | F | df | p | f^2^ |
| Type | 1.478 | 3.000 | 0.249 | 0.163 | 0.918 | 3.000 | 0.450 | 0.084 | 1.470 | 3.000 | 0.253 | 0.137 |
|  | Estimate | SE | p | g | Estimate | SE | p | g | Estimate | SE | p | g |
| (Intercept) | 0.028 | 0.008 | 0.002 |  | 0.022 | 0.008 | 0.010 |  | -0.013 | 0.007 | 0.064 |  |
| R | -0.025 | 0.016 | 0.120 | **-0.890** | -0.018 | 0.014 | 0.214 | -0.546 | 0.005 | 0.012 | 0.658 | 0.073 |
| H | -0.008 | 0.016 | 0.626 | -0.540 | -0.012 | 0.014 | 0.399 | **-1.001** | 0.020 | 0.012 | 0.124 | **1.020** |
| CB | -0.036 | 0.020 | 0.089 | -0.056 | -0.022 | 0.019 | 0.256 | 0.130 | 0.027 | 0.016 | 0.112 | 0.471 |
| **B** | Estimate | SE | p | f^2^ | Estimate | SE | p | f^2^ | Estimate | SE | p | f^2^ |
| (Intercept) | 0.004 | 0.080 | 0.960 |  | -0.011 | 0.053 | 0.851 |  | 0.010 | 0.068 | 0.891 |  |
| Age | -0.001 | 0.004 | 0.762 | 0.026 | -0.001 | 0.002 | 0.707 | 0.041 | 0.001 | 0.003 | 0.805 | 0.017 |
| **C** | Estimate | SE | p | f^2^ | Estimate | SE | p | f^2^ | Estimate | SE | p | f^2^ |
| (Intercept) | -0.087 | 0.125 | 0.523 |  | -0.104 | 0.079 | 0.258 |  | 0.049 | 0.109 | 0.674 |  |
| Width | 0.001 | 0.002 | 0.614 | 0.075 | 0.001 | 0.001 | 0.400 | 0.221 | 0.000 | 0.002 | 0.845 | 0.011 |
| F=F value, df=degrees of freedom, p=p-value at 95% CI, SE=standard error, g=Hedges’s g, f^2^=Cohen’s f^2^ | | | | | | | | | | | | |

**Supplementary Table S8.** Results for linear mixed model (y ~ Type + 1|GroupID) (A) and simple regressions using *Age* (B) and *Width* (C) as predictors. Response variables (Fst and G”st) are estimators of genetic differentiation between populations. A population was defined as a set of sampled individuals on same side of the barrier within each plot. For *Age* and *Width* models, response variables were computed as differences (Δ) between experimental and their associated controls for 4-lane highways, exclusively (see text for further details). GroupID: a unique identifier for each pair of experimental/control plots. Intercept in A represents the level taken as reference for contrasts (unpaved rural tracks, T). Large effect sizes are highlighted in bold.

| **Fst** | | | | | **G”st** | | | | |
| --- | --- | --- | --- | --- | --- | --- | --- | --- | --- |
| **A** | F | df | p | f^2^ | | F | df | p | f^2^ |
| Type | 1.790 | 3.000 | 0.182 | 0.205 | | 0.672 | 3.000 | 0.579 | 0.077 |
|  | Estimate | SE | p | g | | Estimate | SE | p | g |
| (Intercept) | -4.430 | 0.102 | <0.001 |  | | 0.015 | 0.007 | 0.033 |  |
| R | 0.278 | 0.176 | 0.131 | 0.613 | | 0.009 | 0.012 | 0.465 | 0.567 |
| H | -0.177 | 0.176 | 0.327 | -0.439 | | -0.008 | 0.012 | 0.481 | -0.391 |
| CB | -0.305 | 0.236 | 0.211 | -0.312 | | -0.015 | 0.016 | 0.347 | **-0.924** |
| **B** | Estimate | SE | p | f^2^ | | Estimate | SE | p | f^2^ |
| (Intercept) | -0.001 | 0.008 | 0.880 |  | | -0.017 | 0.032 | 0.611 |  |
| Age | 0.000 | 0.000 | 0.955 | 0.001 | | 0.000 | 0.001 | 0.865 | 0.008 |
| **C** | Estimate | SE | p | f^2^ | | Estimate | SE | p | f^2^ |
| (Intercept) | 0.002 | 0.013 | 0.868 |  | | -0.034 | 0.050 | 0.535 |  |
| Width | 0.000 | 0.000 | 0.764 | 0.026 | | 0.000 | 0.001 | 0.679 | 0.049 |
| F=F value, df=degrees of freedom, p=p-value at 95% CI, SE=standard error, g=Hedges’s g, f^2^=Cohen’s f^2^ | | | | | | | | | |

**Supplementary Table S9.** Description of type of barrier (R> conventional 2-lane roads, H> 4-lane highways, and CB> combined barriers), main features (width -in meters- and years built) and geographic location of experimental and control sites (plots). See also Figure 1.

| **Plot** | **Type** | **Locality** | **Lat.** | **Long.** | **Barrier width** | **Barrier age** |
| --- | --- | --- | --- | --- | --- | --- |
|  |  |  |  |  |  |  |
| CB1 | CB | Marcilla de Campos | 42.34 | -4.39 | 100.00 | 9 |
| CB1c | Control | Osornillo | 42.35 | -4.31 | 4.14 | -- |
| CB2 | CB | Villaherreros | 42.38 | -4.52 | 204.60 | 15 |
| CB2c | Control | Bahillo | 42.43 | -4.60 | 4.16 | -- |
| CB3 | CB | San Nicolás del Real Camino | 42.37 | -4.96 | 133.36 | 16 |
| CB3c | Control | Población de Arroyo | 42.33 | -4.89 | 5.79 | -- |
| H1 | H | El Burgo Ranero | 42.42 | -5.28 | 42.40 | 20 |
| H1c | Control | Villamuñió | 42.48 | -5.26 | 5.83 | -- |
| H2 | H | Bustillo del Páramo de Carrión | 42.35 | -4.71 | 43.01 | 16 |
| H2c | Control | Villarrabé | 42.42 | -4.76 | 4.77 | -- |
| H3 | H | Abia de las Torres | 42.45 | -4.39 | 55.03 | 8 |
| H3c | Control | Castrillo de Villavega | 42.44 | -4.47 | 3.79 | -- |
| H4 | H | Mota del Marqués | 41.62 | -5.16 | 71.74 | 26 |
| H4c | Control | Villalbarba | 41.58 | -5.18 | 3.70 | -- |
| H5 | H | Vega de Valdetronco | 41.58 | -5.10 | 54.15 | 26 |
| H5c | Control | Villalar de los Comuneros | 41.53 | -5.12 | 3.80 | -- |
| H6 | H | Villardefrades | 41.74 | -5.27 | 44.28 | 26 |
| H6c | Control | Villavellid | 41.71 | -5.30 | 4.64 | -- |
| R1 | R | Becilla de Valderaduey | 42.12 | -5.24 | 14.46 | 61 |
| R1c | Control | Villalba de la Loma | 42.16 | -5.19 | 3.94 | -- |
| R2 | R | La Mudarra | 41.81 | -4.99 | 15.69 | 61 |
| R2c | Control | Valdenebro de los Valles | 41.87 | -4.98 | 4.98 | -- |
| R3 | R | Villanueva del Campo | 42.00 | -5.39 | 15.63 | 61 |
| R3c | Control | Villar de Fallaves | 41.93 | -5.37 | 4.65 | -- |
| R4 | R | Matallana de Valmadrigal | 42.38 | -5.34 | 16.40 | 61 |
| R4c | Control | San Román de los Oteros | 42.40 | -5.41 | 3.96 | -- |
| R5 | R | Villamartín de Campos | 42.02 | -4.68 | 13.38 | 61 |
| R5c | Control | Fuentes de Nava | 42.08 | -4.71 | 3.71 | -- |
| R6 | R | Villacid de Campos | 42.08 | -5.08 | 13.07 | 61 |
| R6c | Control | Villabaruz de Campos | 42.02 | -5.01 | 3.96 | -- |


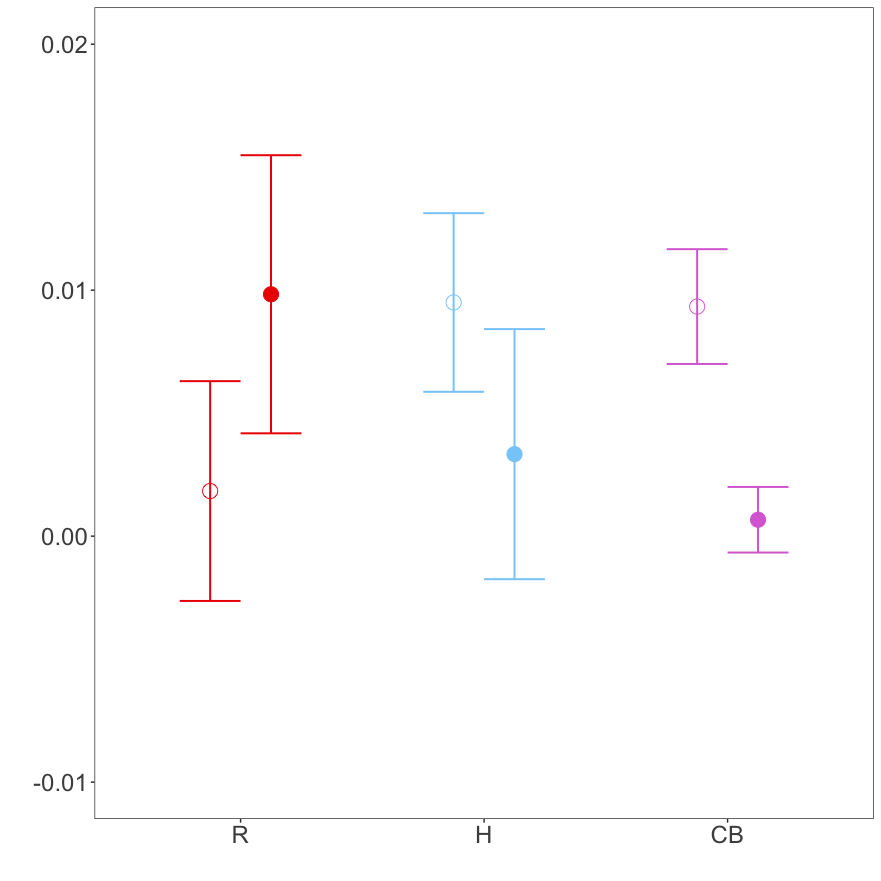

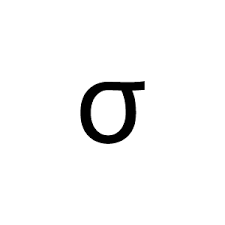


BW

**Supplementary Figure S1.** Mean and standard error for AMOVA results. Y-axis represents the ratio computed dividing σ_Between_ (variance between opposite sides of the barrier) by σ_Within_ (variance within the same side of the barrier). Values are computed for plots belonging to each type of putative barrier (filled dots; R, H, CB) and compared to their paired controls (empty dots).
